# Antibacterial and Antioxidant Activities of Novel *Actinobacteria* Strain Isolated from Gulf of Khambhat, Gujarat

**DOI:** 10.3389/fmicb.2017.02420

**Published:** 2017-12-07

**Authors:** Riddhi N. Dholakiya, Raghawendra Kumar, Avinash Mishra, Kalpana H. Mody, Bhavanath Jha

**Affiliations:** Marine Biotechnology and Ecology Division, CSIR-Central Salt and Marine Chemicals Research Institute, Bhavnagar, India

**Keywords:** *Actinobacteria*, antibacterial, antioxidant, biolog, marine bacteria, novel strain, polyketide synthases, sea sediment

## Abstract

Bacterial secondary metabolites possess a wide range of biologically active compounds including antibacterial and antioxidants. In this study, a Gram-positive novel marine *Actinobacteria* was isolated from sea sediment which showed 84% 16S rRNA gene sequence (KT588655) similarity with *Streptomyces variabilis* (EU841661) and designated as *Streptomyces variabilis* RD-5. The genus *Streptomyces* is considered as a promising source of bioactive secondary metabolites. The isolated novel bacterial strain was characterized by antibacterial characteristics and antioxidant activities. The BIOLOG based analysis suggested that *S. variabilis* RD-5 utilized a wide range of substrates compared to the reference strain. The result is further supported by statistical analysis such as AWCD (average well color development), heat-map and PCA (principal component analysis). The whole cell fatty acid profiling showed the dominance of iso/anteiso branched C15–C17 long chain fatty acids. The identified strain *S. variabilis* RD-5 exhibited a broad spectrum of antibacterial activities for the Gram-negative bacteria (*Escherichia coli* NCIM 2065, *Shigella boydii* NCIM, *Klebsiella pneumoniae, Enterobacter cloacae, Pseudomonas* sp. NCIM 2200 and *Salmonella enteritidis* NCIM), and Gram-positive bacteria (*Bacillus subtilis* NCIM 2920 and *Staphylococcus aureus* MTCC 96). Extract of *S. variabilis* strain RD-5 showed 82.86 and 89% of 2,2-diphenyl-1-picrylhydrazyl (DPPH) free radical scavenging and metal chelating activity, respectively, at 5.0 mg/mL. While H_2_O_2_ scavenging activity was 74.5% at 0.05 mg/mL concentration. Furthermore, polyketide synthases (PKSs types I and II), an enzyme complex that produces polyketides, the encoding gene(s) detected in the strain RD-5 which may probably involve for the synthesis of antibacterial compound(s). In conclusion, a novel bacterial strain of *Actinobacteria*, isolated from the unexplored sea sediment of Alang, Gulf of Khambhat (Gujarat), India showed promising antibacterial activities. However, fractionation and further characterization of active compounds from *S. variabilis* RD-5 are needed for their optimum utilization toward antibacterial purposes.

## Introduction

More than 70% of the surface of the earth planet covers by the sea which contains exceptional diversity which is more than 95% of the whole biosphere (Qasim, 1999). It was observed that the living diversity is higher in some marine ecosystems, such as the deep sea and coral reefs, than the tropical rainforests (Edwards et al., 2006). The ocean is the habitat of several groups of life-forms which live in a complex environment with extreme variations in pressure, salinity, light, and temperature (Munn, 2004). Recently, it was proven that the ocean floor possesses many unique forms of *Actinobacteria* (Fenical and Jensen, 2006). *Actinobacteria* are widely distributed in intertidal zones, mangroves, seawaters, animals, plants, sponges, and in ocean sediments (Goodfellow and Williams, 1983; Castillo et al., 2005; Jensen and Mafnas, 2006; Ramesh and Mathivanan, 2009; Sun et al., 2010; Xiao et al., 2011; Rao and Rao, 2013). *Actinobacteria* from the marine environment are considered as a promising source of pharmaceutically important compounds because of a different kind of unique adaptation characteristics (Fenical and Jensen, 2006; Jose and Jha, 2017).

*Actinobacteria* are Gram-positive bacteria with filamentous structure. These are considered the most economical and biotechnological important prokaryotes which produce several secondary metabolites with significant biological activities. Out of these *Actinobacteria, Streptomyces* is an important industrial group of organisms that widely explored for the wide range of biologically active compounds (Berdy, 2005). *Actinobacteria* comprise of G + C rich microorganisms (Embley and Stackebrandt, 1994), live in varying habitats and well established for the synthesis of bioactive secondary metabolites (Sengupta et al., 2015). *Actinobacteria* inhabiting marine environment (such as sea sediments, etc.) gain much attention (Lane and Moore, 2011) because they are considered more challenging to culture compared to their terrestrial relatives. They have special growth requirements and media composition. Furthermore, several *Actinobacteria* genera produce novel secondary metabolites with several bioactivities (Jensen et al., 2005). The recent grasp of the fact that marine environment can be a potential source for the novel isolates with novel natural products encourages intensive search and efforts from several groups. Nearly seventy five percent of all the known industrial antibiotics (Kieser et al., 2000) and numerous economically important compounds (Okami and Hotta, 1988) were obtained from the streptomyces’s. *Actinobacteria* have also ability to synthesize antiviral (Sacramento et al., 2004), antifungal (Zarandi et al., 2009), antitumor (Hong et al., 2009), insecticidal (Pimentel-Elardo et al., 2010), antioxidants (Janardhan et al., 2014), anti-inflammatory (Renner et al., 1999), anti-biofouling (Xu et al., 2010), immunosuppressive (Mann, 2001), anti-parasite (Pimentel-Elardo et al., 2010), plant growth promoting and herbicidal compounds (Sousa et al., 2008), enzyme inhibitors (Hong et al., 2009) and industrially important enzymes. Advance molecular tools such as metagenomics, metatranscriptomics, and metaproteomics can be employed directly for the extraction of DNA, RNA, and protein from environment samples (Mincer et al., 2005). Simultaneously, polymerase chain reaction (PCR) amplified products were cloned and sequenced for identifying new *Actinobacteria* present in the environment samples (Monciardini et al., 2002; Riedlinger et al., 2004). Selective primer is now available to amplify the 16S rRNA gene from the specific *Actinobacteria* (Monciardini et al., 2002). Metabolic bioactive compounds obtained from marine or territorial *Actinobacteria* are commonly synthesized by enzymes polyketide synthases (PKS) or non-ribosomal peptide synthetases (NRPS). The PKS is categorized into three different groups such as types I, II, and III. Both NRPS peptides and PKS-type I are encoded by a number of modules which are multifunctional in nature (Ayuso-Sacido and Genilloud, 2005; Smith and Sherman, 2008). They form a series of biosynthesis reaction including acyl (PKS-I) or peptidyl (NRPS) chain initiation, elongation, and termination (Walsh, 2008). PKS-II molecules which are non-modular, complex of several single module proteins and their group of enzymatic activity act in an iterative manner to produce a polyketide (Gallo et al., 2013). The core PKS module comprises of a ketoacyl-synthase (KSα), a chain elongation factor (KSβ), and an acyl-carrier protein (Walsh, 2004; Das and Khosla, 2009). The PKS-III types are homodimer enzymes and act on the acyl-CoA without involving any acyl-carrier proteins (Shen, 2003). In continues searching potential bioactive, molecular methods will help for analyzing and comparing the genetic variations within these genes, in the normal laboratory condition strain’s specialized metabolites is not routinely produced which are useful for screening for molecule production is remains mostly a trial-and-error approach (Metsa-Ketela et al., 1999; Ayuso-Sacido and Genilloud, 2005; Gontang et al., 2010).

Extensive study has been done on various coastal areas of India for isolation and cultivation of *Actinobacteria*. However, the coast of Gujarat and especially, Gulf of Khambhat is relatively unexplored so far. Therefore, the present study was aimed to investigate the novel marine *Actinobacteria* using molecular methods and phylogenetic comparisons of the isolates. Furthermore, the isolated bacterial strain was functionally characterized by antibacterial and antioxidant activities. The present study provides a useful insight of bacteria inhabiting sea sediment of Arabian Sea. The isolated bacterial strain can be utilized further for the developing novel antibacterial compounds.

## Materials and Methods

### Isolation and Culture Characterization of Marine *Actinobacteria*

The sea sediment samples (25 g) were collected from coastal areas of Gulf of Khambhat, Gujarat, India near a ship scraping industries (21°24′35.85″N, 72°11′54.1″E). Samples were transported to the laboratory under cool and control conditions, and immediately processed for the isolation of marine *Actinobacteria* (through serial dilution method) from sediment samples using modified Gause’s Synthetic Agar medium (Ye et al., 2009). In brief, 0.5 g sea sediment was suspended in 9.5 ml of sterile saline solution (0.9% NaCl). The suspended solution was serially diluted up to 10^-10^ in saline solution. About 100 μl diluted solution (10^-3^ to 10^-10^) was spread individually on modified Gause’s Synthetic Agar medium containing 0.01% (w/v) potassium dichromate to prevent the early growth of other bacteria and fungus. Plates were incubated at 30°C for 4–7 days, and *Actinobacteria* were preliminarily screened based on traditional morphology.

### BIOLOG Assay of Selected *Actinobacteria* Isolates

The isolated bacteria were categorized by GEN III MicroPlate test assay performed with a Biolog system. The test panel comprises of 71 carbon sources with 23 chemical sensitivity assays and thus provides a “Phenotypic Fingerprint” of the tested microorganism. The Biolog system dissects and analyses the ability of a cell to metabolize all major substrates. Furthermore, other important physiological properties such as salt, pH, reducing power, chemical sensitivity and lactic acid tolerance were also determined. Overnight grown bacterial suspensions were mixed with 0.85% saline solution (5 mL) and IF-a was adjusted for 90–98% transmittance (T90) with a Biolog turbidimeter. Into each well of Biolog microplate, about 100 μL bacterial suspension was dispensed and incubated at 30°C. The developed color is compared with the Biolog species library to identify the bacterial isolates.

### Average Well Color Development Assay

BIOLOG plates are commonly used for the analysis of microbial community function and micro-organism may be identified by the specific phenotype color fingerprint. The average well color development (AWCD) quantification of individual plate or individual well is performed by continuous monitoring of OD absorbance at 590 nm. The measured data was expressed as AWCD in response to incubation time (Garland and Mills, 1991).

$$\begin{array}{c} \mathrm{AWCD}\;=\;\Sigma ODi/95 \end{array}$$

### Chemotaxonomic Identification

Chemotaxonomic identification of isolates was done by fatty acid methyl ester (FAME) analysis using gas chromatography coupled with Sherlock microbial identification system (MIS). The MIS gives the data output includes fatty acids composition and sample chromatographic run. The software computes “Sim index” which congregates values of samples FAME with the library and gives a Euclidian distance (ED).

### Molecular Identification

Isolate RD-5 was grown in 50 mL of Gause’s Synthetic broth containing NaCl (4%, w/v) for 7 days. The mycelia were harvested by centrifugation at 10,000 rpm for 5 min and genomic DNA was extracted using phenol-chloroform extraction method (Hopwood et al., 1985). DNA quality and concentration were measured using a Nanodrop 1000 Spectrophotometer.

The 16S rRNA gene was amplified using genomic DNA and universal bacterial primers (**Table 1**). The 50 μL PCR mixture was contained; 1–2 μL DNA template, 0.5 μL 20 μM of each primer, 5 μL of 10X buffer, 5 μL of dNTPs (2.5 mM), 0.5 μL Taq DNA (5 units/μL), and 41.5 mL ddH_2_O. PCR was done in MyCyclerT-100 (Bio-Rad, United States) using the optimized conditions (Yousuf et al., 2012, 2014a,b; Keshri et al., 2013; Keshri et al., 2015). The amplified products were analyzed on a 1.0% agarose gel, purified (QIAquick PCR Purification Kit, Qiagen, Germany) and sent to M/s Macrogen, S. Korea for the sequencing services. The 16S rRNA gene sequence was aligned using BioEdit software, compared with gene sequences available in the databases (NCBI + DDBJ + EMBL) and deposited in GenBank with an accession number KT588655. The putative phylogenetic affiliation was determined using the naïve Bayesian rRNA classifier and RDP-II database with the 95% confidence (Wang et al., 2007; Cole et al., 2009).

**Table 1:** List of primers used for amplification of non-ribosomal peptide synthetases (NRPS) and PKS-1gene fragments and 16S rRNA.

Primer Name	DNA sequences (5′–3′)	Name of product	Target size	Reference
27F 1492R	5′- AGAGTTTGATCMTGGCTCAG -3′ 5′- ACCTTGTTACGACTT -3′	16S rRNA	1.5 Kb	Lane, 1991
K1F M6R	5′-TSAAGTCSAACATCGGBCA-3′ 5′-CGCAGGTTSCSGTACCAGTA-3′	Type-I polyketide synthases (PKS-I)	1.4 Kb	Ayuso-Sacido and Genilloud, 2005
KSαF KSαR	5′-TSGCSTGCTTGGAYGCSATC-3′ 5′-TGGAANCCGCCGAABCCTCT-3′	Ketosynthase gene (PKS-II)	700 bp	Metsa-Ketela et al., 1999

### Bioactivity from Marine *Actinobacteria*

#### Primary Screening of Marine *Actinobacteria* for Antibacterial Activity

The isolated and purified *Actinobacteria* isolates were screened for antibacterial activity by cross streak method (Balagurunathan and Subramanian, 2001) using Mueller-Hinton agar (Himedia, India) against eight different pathogenic bacteria; Gram-negative (*Escherichia coli* NCIM 2065, *Shigella boydii* NCIM, *Klebsiella pneumonia, Enterobacter cloacae, Pseudomonas* sp. NCIM 2200, *Salmonella enteritidis* NCIM, and two Gram-positive bacteria (*Bacillus subtilis* NCIM 2920 and *Staphylococcus aureus* MTCC 96). Plates containing well grown RD-5 strain was cross streaked with pathogenic bacteria at 90° angles and incubated at 37°C overnight. Antagonism was observed by noting the absence or presence of pathogenic bacterial growth.

#### Optimization of Growth Conditions for the Production of Bioactive Compounds

The promising strain RD 5 was cultured in six different media; starch casein agar, yeast malt extract agar (ISP2), glycerol asparagine agar (ISP5), inorganic salt agar (ISP-4), tyrosine agar (ISP-7) and gause’ synthetic agar (GSA) and incubated at 30°C for 7–9 days. The cell mass was measured by the dry weight of cell biomass after 24 h interval and compound production was measured using well diffusion method at 24 h interval for 9 days. The experiments were repeated three times for each assay.

#### Extraction of Bioactive Compounds and Bioactivity Assay

The most promising isolate (RD-5) was grown in the optimized gause’s synthetic broth (GSB) media for the isolation of the active compounds. The selected isolate was inoculated in GSB medium and incubated for 7 days in shaking condition at 180 rpm at 30°C. Culture media was harvested every 24 h, centrifuged for 15 min at 8,000 rpm and collected supernatant was mixed with an equal volume of ethyl acetate followed by extraction with separating funnel (Ismail et al., 2009). The crude extract was obtained by removing the solvent using rotary evaporator. The dried crude extract was dissolved in methanol, and stock concentration was prepared as 100 mg/mL. The crude extracts (3 to 7 mg) were used for the bioactivity against different pathogenic bacteria using well diffusion method with Mueller Hinton agar (Nandhini and Selvam, 2011). Methanol used as a control and the bioactivity of extracts was noted based on the zone of inhibition. Furthermore, the bacterial extract was evaluated for the different antioxidant and radicals scavenging activity.

#### DPPH Radicals Scavenging Assay

2,2-diphenyl-1-picrylhydrazyl (DPPH) radical scavenging activity of the bacterial extract was determined using method reported by (Bersuder et al., 1998) using different concentrations of melanin (0.05–5.0 mg/mL). In test tubes, different concentration of melanin was taken, and volume was made up to 2 mL with distilled water. About 2 mL of 0.002% DPPH solution was added to each tube, mixed and incubated for 30 min in the dark. Reduction of DPPH radical was quantified at 517 nm using UV-Vis spectrophotometer. The percentage of DPPH radical scavenging activity was calculated as:

$$\begin{array}{c} \mathrm{DPPH}\mathrm{radical}\mathrm{scavenging}\mathrm{activity}[\%]\;=\;[(A_{c}\;-\;A_{s})/A_{c}]\;\times \;100 \end{array}$$

Where, *A*_c_ and *A*_s_ were the absorbance of the control and sample, respectively. The experiment was conducted in triplicates.

#### Hydrogen Peroxide Radical Scavenging Activity

A solution of hydrogen peroxide (40 mmol/L) was prepared in phosphate buffer (pH 7.4). To 4 mL of bacterial extract of different range of concentrations (0.05–5.0 mg/mL), 0.6 mL of H_2_O_2_ solution was added. The absorbance was measured at 230 nm by the UV-visible spectrometer and percentage inhibition of H_2_O_2_ scavenging activity was calculated (Keser et al., 2012; Mishra et al., 2015; Patel et al., 2016).

$$\begin{array}{c} H_{2}O_{2}\mathrm{scavenging}\mathrm{activity}[\%]\;=\;[(A_{c}\;-\;A_{s})/A_{c}]\;\times \;100 \end{array}$$

Where *A*_c_ and *A*_s_ were the absorbance of control and test samples, respectively. The experiment was conducted in triplicates.

#### Metal Chelating Activity

The ferrous ions chelating activity of the bacterial extract was analyzed (Dinis et al., 1994). Different concentration of extract (0.05–5.0 mg/mL) was made up with final volume 0.5 mL and mixed with 0.05 mL of 2 mM FeCl_2_. About 0.2 mL ferrozine solution (5 mM) was added to the reaction mix, shaken vigorously and kept for 10 min at room temperature. The absorbance of the reaction mix was estimated at 562 nm and percentage inhibition of ferrozine-Fe^2+^ complex formations was calculated:

$$\begin{array}{c} \%\;\mathrm{of}\;\mathrm{inhibition}\;=\;(A_{s}/A_{c})\;\times \;100 \end{array}$$

Where *A*_c_ and *A*_s_ were the absorbance of control and test samples, respectively.

#### Cloning of PKS-I and PKS-II Genes

Two set of degenerative primers were designed to amplify internal fragment of KSα and PKS-I biosynthetic genes fragments from RD-5 strain (**Table 1**). PCR was done in 25 μl volume that contained 1X Taq buffer, 2.5 μL of dNTPs (2.5 mM), 20 pM primers (forward and reverse), 0.05 U of Taq DNA polymerase enzyme (Sigma, United States) and 10–15 ng genomic DNA. PCR was carried out with denaturation of the templete DNA at 95°C for 5 min followed by 35 cycles at 95°C for 30 s, primer annealing at 58°C for 120 s, for the KS of PKS-II domain while 55°C for 2 min was used for the amplification of K1F/M6R PKS-I gene and finally extension at 72°C for 4 min. Amplified PCR products were analyzed on 1% agarose gel, purified, cloned into pGEM-T easy vector (Promega, United States) and transformed to *E. coli* DH5α. Recombinant plasmid DNA was extracted using alkaline lysis method and confirmed by PCR with vector-specific primers M13F and M13R. Both cloned genes fragments, PKS-1 and PKS-II were sequenced from M/s Macrogen Inc, South Korea and deposited in GenBank with the accession numbers MG459176 and MG459177, respectively.

### Phylogenetic Analysis

The 16S rRNA gene sequences (KT588655) were subjected to BLASTn for the comparision with the other 16S rRNA gene sequences exist in GenBank and closest relative 16S ribosomal RNA sequences were retrieved from NCBI database (Zhang et al., 2000). Sequence alignment was performed with cluster W (Altschul et al., 1997), phylogenetic trees were constructed (using Mega ver. 6) with the neighbor-joining method and a bootstrap value of 1000 replicates (Tamura et al., 2013). The resultant sequence of both PKS-I and PKS-II genes fragment was also analyzed with BLASTx search and protein sequence were retrieve from NCBI, aligned and the phylogenetic tree was constructed using the neighbor-joining tree-making algorithm.

### Statistical Analysis

Average well color development (AWCD), diversity richness (R), and Shannon evenness (E) were calculated by analysis of variance (ANOVA) of each strain based on color development with every 24 h. The cluster analysis was used to evaluate the most utilized substrate for each strain. The AWCD data was standardized to remove inoculum density effects. Ordination methods were used for principal component analysis (PCA) of the data taken at 96 h. The method categeorised samples on scatter plots of two or more axes and the most closest micro-organism come together (Randerson, 1993; Podani, 2000). For the comparision of numerical responses in the 95 substrates, PCA plot reduced the multivariate data set (variables or individuals) and exhibited any changes in the variation of the data.

## Results

### Isolation and Characterization of Actinobacterial Strains

A total of 11 different strains of *Actinobacteria* were isolated from Gulf of Khambhat, Alang, Bhavnagar, Gujarat. The distinctly different isolates based on their morphological and pigmentation were purified by repeated streak method on Gause’s Synthetic Agar medium and preserved at 4°C as on slant. All the isolates were screened with preliminary cross streak assay. Out of them, Isolate RD-5 was found novel, additionally exhibits potent activity against pathogenic bacteria.

**FIGURE 1:**
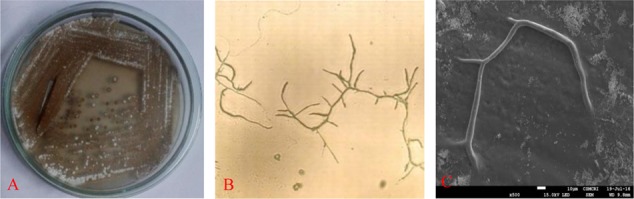
Isolated colonies on **(A)** gause’ synthetic agar (GSA) medium, **(B)** microscopy and **(C)** SEM image of RD-5.

Selected strain was aerobic, Gram-positive and the colonies are dry, powdery, fuzzy with a concentric ring on agar surface which showed secondary metabolite production with diffusible brownish pigment were initially identified as *Actinobacteria* (**Figure 1A**). Microscopic examination of the strain was undertaken under a compound microscope. The short branched vegetative hyphae and aerial mycelia were sparse with a patchy distribution (**Figures 1B,C**).

### Characterization of Microbial Strain(s) from the Selected Cultures Based on BIOLOG

All *Actinobacteria* were examined using Biolog System to obtain their metabolic profiles or biotyping. Biolog System analysis is based on carbon (C) utilization patterns of the *Actinobacteria* toward different carbon source. The ability to use a wide range of carbon source may indicate that the *Actinobacteria* were able to survive in the different environment in nature. Metabolic profiles resulted from Biolog GENE III System analysis indicated the 11 *Actinobacteria* were differentiated into different strains. As shown in (Supplementary Table S1) the eleven strains of *Actinobacteria* (RD-1 to RD-9 and RD 15 and RD 16) have the different capability to metabolize 95 carbon sources from GENE III microplates. The 95 carbon sources are categorized as polymers, sugar and sugar derivatives, carboxylic acids and methyl esters, carboxylic acids and methyl esters, alcohol, nucleosides and nucleotides and sugar phosphates. Of the 95 carbon sources, only 75 can be utilized by the all eleven strains of *Actinobacteria*. Strain RD-5 was one from the eleven colonies, showing significantly higher in carbon sources activity with 90 substrates followed by Strain RD-6 and RD-9 using 89 followed by RD-4, and RD-16 using 88 RD-15, RD-8 and RD-1, RD-2 and RD-7 and RD-3 with 87, 86, 82, and 81, respectively.

### Monitoring Color Development in BIOLOG^TM^ GENE III Plates with Other Reference Strain of *Actinobacteria*

**FIGURE 2:**
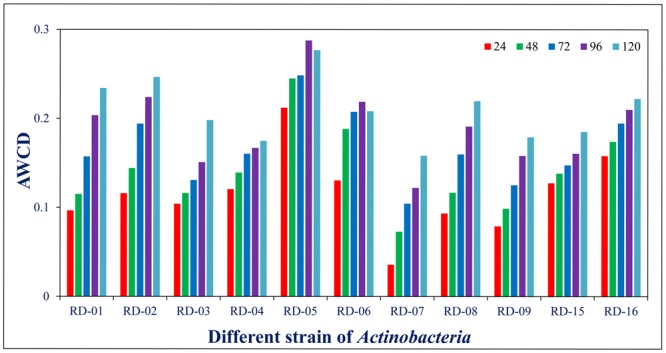
Average well color development (AWCD) of metabolized substrates in BIOLOG GENE III in every 24 h.

**FIGURE 3:**
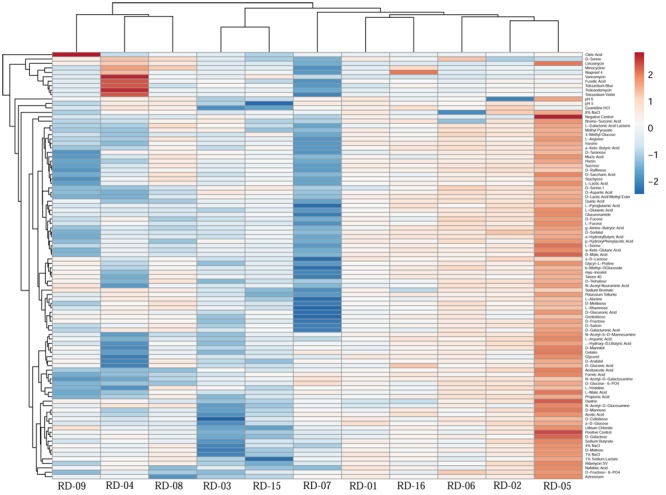
This cluster heat map was generated using the http://biit.cs.ut.ee/clustvis/ online program package with Euclidean distance as the similarity measure and hierarchical clustering with complete linkage.

**FIGURE 4:**
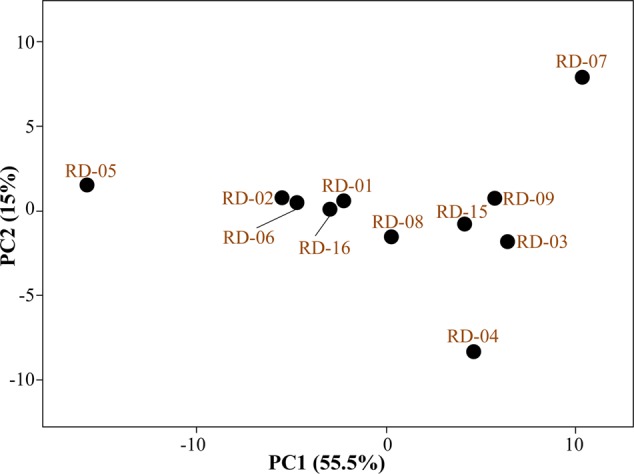
Principal component analysis (PCA) of all 11 strain of *Actinobacteria.*

Normalized value of AWCD further evidence that different strain cluster (**Figure 2**). In the hierarchical clustering with the complete linkage, RD-5 shows the most of the substrate is utilized in 96 h, but other strain is less used the substrate (**Figure 3**). PCA of ordinance methods scatter plot of each strain in BIOLOG allow the sample to be represented two or more axis PC1 (55.5%) second one PC2 (15%) RD-5 was scatter in PC2 axis (**Figure 4**).

### FAME Analysis

**FIGURE 5:**
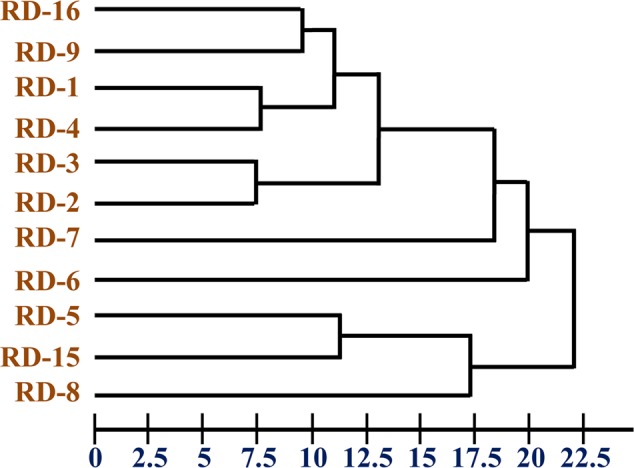
Dendrogram of fatty acid methyl ester (FAME) profiles novel strain RD-5 with reference strain.

The chemotaxonomic study of the potential isolate RD-5 revealed that it belongs to the *Actinobacteria*. Saturated iso/anteiso- branched fatty acids with C15–C17 long chain was detected as major cellular fatty acids. The cluster analysis of FAME profile showed correlation among organisms by Euclidian distance. Cluster containing isolates identified was delineated at 22.5 ED (**Figure 5**) were closely matched those of *Streptomyces*, but considerable differences were recorded among the eleven strains.

### Phylogenetic Analysis of 16S rRNA

**FIGURE 6:**
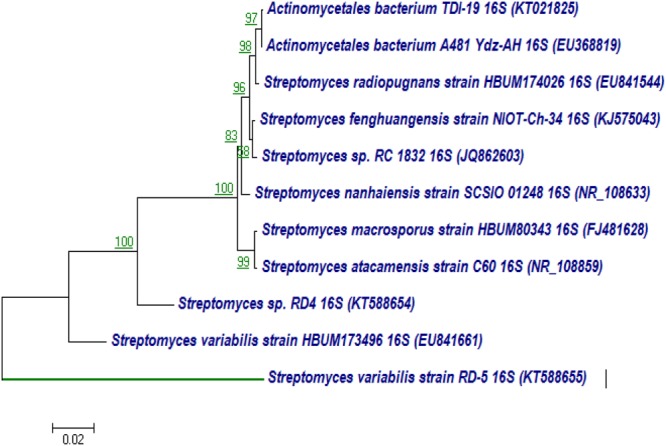
Neighbor-joining tree based on nearly complete 16S rRNA gene sequences showing relationships between strain RD-5 and closely related members of the genus *Streptomyces*. Numbers at nodes indicate levels of bootstrap support based on a neighbor-joining analysis of 1000 resampled datasets; only values above 50% are given. Bar, 0.02 substitutions per nucleotide position.

**FIGURE 7:**
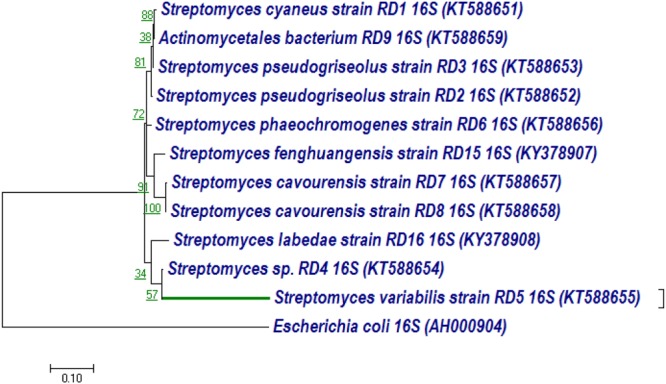
Neighbor-joining tree based on nearly complete 16S rRNA gene sequences showing relationships between strain RD-5 and closely related members of the genus *Streptomyces* as reference strain with out-group *Escherichia coli*. Numbers at nodes indicate levels of bootstrap support based on a neighbor-joining analysis of 1000 resampled datasets; only values above 50% are given. Bar, 0.10 substitutions per nucleotide position.

The 16S rRNA gene of RD-5 was amplified and sequenced (KT588655). The partial 16S rRNA gene sequence of RD-5 covered a stretch of 1382 bp having an average 54.8% G+C content. Nucleotides were subjected to BLASTn analysis (**Table 2**) which showed the 84% similarity with *Streptomyces variabilis*. The nucleotide sequences of the type strain were retrieved from the NCBI, and a phylogeny was studied (**Figure 6**). The phylogenetic position of the strain was within a cluster that contains *Streptomyces fenghuangensis* (KJ575043), *Actinomycetales bacterium* (KT021825), and *Streptomyces* sp. RD-4 (KT588654). *Streptomyces* sp. RD-5 was posed with as single branch and shared with 99% Query cover and 82% sequence identity with a closed group. Another phylogenetic tree was constructed with the reference strain, and out-group were taken *E. coli*, and it does not show any similarity match with reference strain (**Figure 7**).

**Table 2:** The BLASTn results, of 16S rRNA according to the NCBI database.

Description	Accession number	Maximum query cover	Maximum score	Total score	Maximum identity (%)
*Streptomyces variabilis* strain RD-5 16S ribosomal RNA gene, partial sequence	KT588655.1	100%	2553	2553	100%
*Streptomyces variabilis* strain HBUM173496 16S ribosomal RNA gene, partial sequence	EU841661.1	99%	1299	1299	84%
*Streptomyces variabilis* strain 173634 16S ribosomal RNA gene, partial sequence	EU570414.1	99%	1085	1085	81%
*Streptomyces variabilis* strain 173500 16S ribosomal RNA gene, partial sequence	EU570413.1	99%	1055	1055	81%
*Streptomyces* sp. RD4 16S ribosomal RNA gene, partial sequence	KT588654.1	99%	1168	1168	82%
*Streptomyces fenghuangensis* strain NIOT-Ch-34 16S ribosomal RNA gene, partial sequence	KJ575043.1	99%	1142	1142	82%
*Streptomyces radiopugnans* strain HBUM174024 16S ribosomal RNA gene, partial sequence	EU841699.1	99%	1127	1127	82%
*Streptomyces nanhaiensis* strain JA 24 16S ribosomal RNA gene, partial sequence	KJ947850.1	94%	1050	1050	81%
*Streptomyces atacamensis* strain C60 16S ribosomal RNA gene, partial sequence	NR_108859.1	99%	1092	1092	81%

These 16S rRNA sequences were also classified in Rdp Naive Bayesian rRNA Classifier Version 2.11 database with >1200 Nucleotide and Confidence threshold is 95% it shows domain Bacteria unclassified_Actinomycetales at the genus level.

### Bioactivity from Marine *Actinobacteria*

#### Primary Screening of Antibacterial Activity

Isolated different marine *Actinobacteria* were primarily screened with the cross streak method for bioactivity against pathogenic bacteria. On the basis of maximum inhibition of pathogenic strain, RD-5 was selected for the further screening.

#### Culture Media Study and Optimization of Cell Growth and Production of the Compound

**FIGURE 8:**
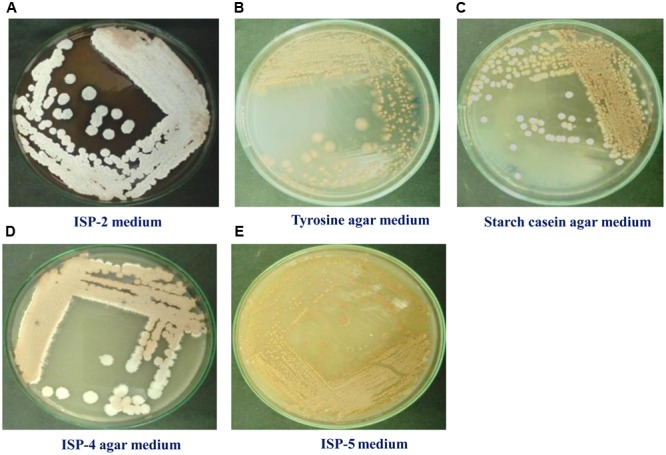
Growth of RD-5 in different media such as **(A)** ISP-2 medium, **(B)** Tyrosine agar medium, **(C)** Starch casein agar medium, **(D)** ISP-4 agar medium and **(E)** ISP-5 medium.

**FIGURE 9:**
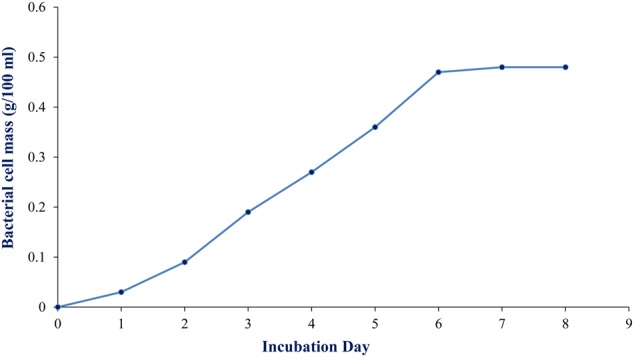
Growth curve of RD-5 at the indicated times, mycelial pellets were harvested for growth determination by biomass measurement.

To maximize the antibacterial production as well as cell mass, strain RD-5 was cultured in five different media, out of six different media, GSA medium supposed to maximize the cell mass as (**Table 3** and **Figure 8**) well as the production of antibacterial activity. The growth curve for strain RD-5 and the antibacterial activity produced in the GSA medium was measured every 24 h of the interval (**Figure 9**). Strain RD-5 showed the first phase of growth 72 h post inoculation. The second phase occurred during 168 h, and thereafter stationary phase occurred. This strain produced compounds after around 72 h and production increased depending on cell growth. The compounds produced were maximized at the end of the second phase. *Shigella boydii* and *Klebsiella pneumonia* showed maximum antibacterial activity from an extract of RD-5 which was 27 mm in both. While the response of *Pseudomonas* sp. was less compared to other pathogenic strains (19 mm).

**Table 3:** Cultural characteristics of *Streptomyces variabilis* RD-5 on different media.

Medium	Growth	Aerial mycelium	Substrate mycelium	Pigment
Starch casein agar	Moderate	Brownish white	Brownish white	None
Yeast malt extract agar (ISP2)	Good	White	Brownish white	Yellow
Inorganic salt agar (ISP-4)	Good	Brownish white	Brownish white	Yellow
Glycerol asparagine agar (ISP5)	Good	Slight orange	Light brown	Light yellow
Tyrosine agar (ISP-7)	Moderate	Light brown	Brownish white	None

#### Secondary Screening of Antibacterial Compound

**FIGURE 10:**
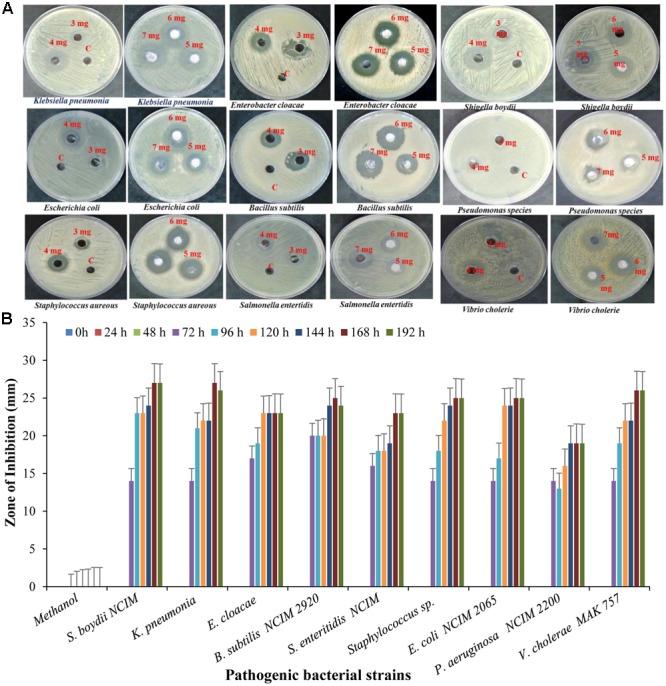
Antibacterial activity of crude extract against different pathogenic strain with **(A)** different concentration and **(B)** different time interval.

The bioactive compounds were extracted from the fermented broth using ethyl acetate solvent, and concentrated crude extract which was used as test compound was carried out by agar well diffusion method. The antibacterial activity of crude extract at concentration of 5 mg/well was assayed against pathogenic strain *Shigella boydii* (13 mm), *Klebsiella pneumonia* (24 mm), *Enterobacter cloacae* (16 mm), *Bacillus pumilus* (22 mm), *Salmonella enteritidis* (14 mm), *Staphylococcus* sp. (16 mm), *E. coli* (15 mm), *Pseudomonas* sp. (17 mm) (**Figure 10**).

#### Antioxidant and Scavenging Activity

**FIGURE 11:**
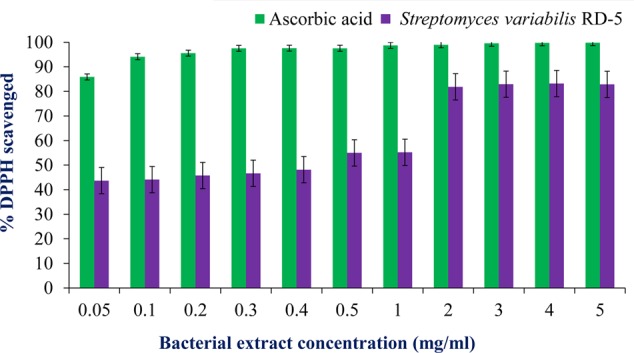
Antioxidant activity of bacterial extract with different concentration.

**FIGURE 12:**
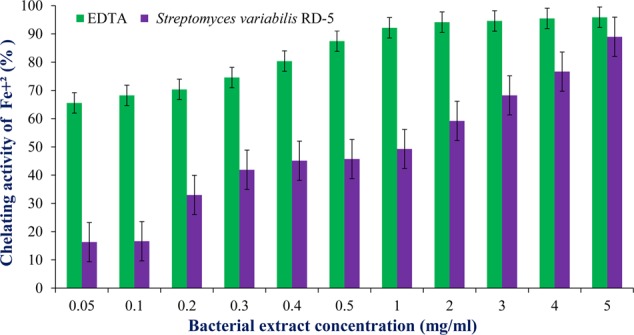
Chelating activity of bacterial extract with different concentration.

**FIGURE 13:**
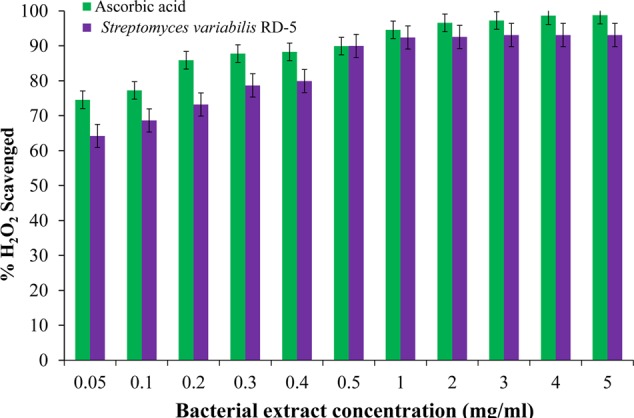
H_2_O_2_ scavenged activity of bacterial extract with different concentration.

DPPH is a stable free radical having absorption maxima at 517 nm. The results of DPPH radical scavenging activity of ethyl acetate extract of *S. variabilis* is depicted in (**Figure 11**). Bacterial extract showed 43.67–82.86% DPPH free radical scavenging activity at 0.05–5.0 mg/mL as compared to ascorbic acid which showed 86% activity at 0.05 mg/mL concentration. The activity of the extract was increased with an increase in concentration and reached to around 55% at 1.0 mg/mL concentration against 98% of ascorbic acid. Further, increase in concentration marginally influenced activity. It was observed that extract of *S. variabilis* showed maximum activity at 2 mg/mL concentration after that slight difference was observed. The activity of the extract increased up to 2.0 mg/mL concentration, a further increase in concentration did not influence activity. Metal chelating activity of extracts of various *Actinobacteria* ranged from 16% as compared to Na-EDTA which showed 65% activity at 0.05 mg/mL concentration (**Figure 12**). With an increase in concentration of extract, the activity increased to 16–89% at 0.05–5 mg/mL while in case of Na-EDTA, 0.5 mg/mL, at concentration yielded 87.5% metal chelating activity. Here, *S. variabilis* exhibited maximum activity at 5 mg/mL concentration. H_2_O_2_ scavenging activity of extracts ranged from 64% as compared to ascorbic acid exhibiting 74.5% activity at 0.05 mg/mL concentration (**Figure 13**). As far as H_2_O_2_ scavenging activity is concerned, extract of *S. variabilis* exhibited activity almost at par with that of ascorbic acid.

#### Phylogenetic Analysis PKS-I and PKS-II Genes

**FIGURE 14:**
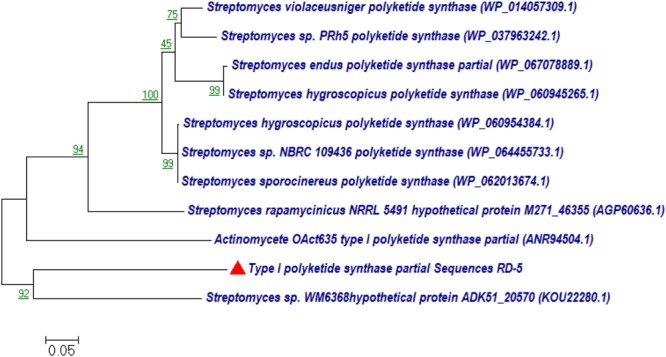
Representative neighbor-joining tree of PKS-I amino acid sequences. The scale bar indicates the number of substitutions that occur per site.

**FIGURE 15:**
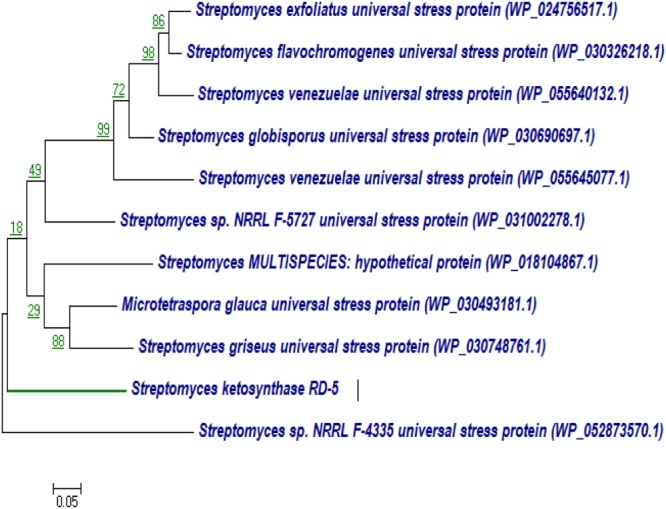
Representative neighbor-joining tree of PKS-II amino acid sequences. The scale bar indicates the number of substitutions that occur per site.

BLASTx analysis of PKS-I and PKS-II amino acid biosynthetic genes of strain RD-5 showed the 99–92% of query cover and 54–52% sequences identity with their closest matches (**Tables 4, 5**). The phylogenetic tree was inferred by maximum likelihood method using the amino acid sequences of both PKS-I and PKS-II of RD-5 novel strain. PKS-I gene sequences showed the maximum identity with *S. hygroscopicus, Streptomyces* sp. NBRC 109436, *S. atratus, S. melanosporofaciens*, and *S. atratus* with 59–56% identity (**Figure 14**). The PKS-II gene sequences showed the maximum identity of 71–49% with previously reported sequences. Amino acid search analysis showed similarity with universal stress and hypothetical proteins from *Streptomyces* sp. NRRL F-5727 (WP_031002278.1), *S. globisporus* (WP_030690697.1), *S. exfoliates* (WP_024756517.1), *S. laurentii* (BAU87338.1), and *Streptomyces* sp. CcalMP-8W (WP_018491225.1) (**Figure 15**).

**Table 4:** The BLASTx results, of PKS-I according to the NCBI database.

Description	Accession no.	Maximum query cover	Maximum score	Total score	Maximum identity (%)
Polyketide synthase (*Streptomyces hygroscopicus*)	WP_060954384.1	99%	419	809	56%
Polyketide synthase (*Streptomyces* sp.) NBRC 109436	WP_064455733.1		418	813	56%
Polyketide synthase 12 (*Streptomyces atratus*)	SFY45126.1	99%	414	709	59%
Type I polyketide synthase (*Streptomyces caatingaensis*)	WP_053161268.1	99%	381	424	55%
type I polyketide synthase (*Streptomyces auratus*) AGR0001	EJJ02441.1	99%	381	514	54%
Type I polyketide synthase 3 (*Streptomyces* sp.)	APD71668.1	99%	379	1103	53%
Beta-ketoacyl synthase (*Streptomyces hygroscopicus*)	WP_078638584.1	99%	392	685	54%
Polyketide synthase 12 (*Streptomyces melanosporofaciens*)	SED16442.1	99%	408	813	55%
Polyketide synthase (*Streptomyces violaceusniger*)	WP_014057309.1	99%	408	817	56%
Polyketide synthase (*Streptomyces hygroscopicus*)	WP_078646099.1	99%	407	808	55%

**Table 5:** The BLASTx results, of PKS-II according to the NCBI database.

Description	Accession number	Maximum query cover	Maximum score	Total score	Maximum identity (%)
Universal stress protein (*Streptomyces* sp.) WM6368	WP_053703232.1	96%	159	159	58%
Universal stress protein (*Streptomyces* sp.) 3211	WP_079403829.1	96%	157	157	58%
Universal stress protein (*Streptomyces* sp. H021)	WP_053631949.1	96%	155	155	57%
Universal stress protein (*Streptomyces virginiae*)	WP_030895366.1	96%	155	155	57%
Universal stress protein (*Streptomyces globisporus*)	WP_030690697.1	92%	188	188	66
Universal stress protein (*Microtetraspora glauca*)	WP_030493181.1	92%	184	184	70%
Universal stress protein (*Streptomyces flavochromogenes*)	WP_030326218.1	92%	173	173	62%
Universal stress protein (*Streptomyces venezuelae*)	WP_055640132.1	92%	171	171	92%
Universal stress protein (*Streptomyces griseus*)	WP_030748761.1	92%	169	169	68%
MULTISPECIES: universal stress protein (*Streptomyces*)	WP_030648525.1	92%	168	168	63%

## Discussion

Adaptations of marine bacteria have developed prodigious metabolic and physiological ability to survive in the extreme conditions that allows them to produce different kind of metabolites, which could not be produced by the terrestrial ones. *Actinobacteria* are well established for producing secondary metabolites with novel antibiotics which are of immense importance to prevent multi-drug resistant pathogens. The *Actinobacteria* produce spores which generally resist desiccation and show to some extent higher resistance toward environmental fluctuation to adopt the harsh condition comparative to others microbes (Hopwood and Wright, 1973).

In the present study, total 11 different isolates were screened, out of them, one promising marine *Actinobacteria* strain, identified as *S. variabilis* RD-5 showed the novelty with antagonistic properties. The phylogenetic position of the *S. variabilis* RD-5 suggested that isolated strain from coastal areas of Gulf of Khambhat have a potential diverse arrangement with novelty which can be useful for many of the applications and can be explored broadly.

Culture medium, GCA, found to be the best for isolation of marine *Actinobacteria S. variabilis* RD-5. The strain showed optimum growth at 30°C on GSA and ISP-2 media. A retarded growth was also showen on Tyrosine agar medium and ISP-5 medium. *S. variabilis* RD-5 was characterized morphologically and microscopically which confirmed its identity as *Streptomyces* genus (Williams et al., 1989; Manfio et al., 1995).

BIOLOG analysis suggested higher AWCS in RD-5 compared to others reference strains of *Actinobacteria*. BIOLOG assay further showed that strain *S. variabilis* RD-5 utilized a wide range of substrates. The cluster and PC analyses showed substrate utilization pattern similar to other *Actinobacteria* community (**Figures 5, 6**). The cluster analysis and PCA showed the comparability of both experiments and further confirmed the overlap metabolic fingerprints among the different strains of *Actinobacteria*.

The16S rRNA gene sequencing and phylogenetic analysis revealed that RD-5 is a novel strain, having identity below 85% as shown by RDP-II classifier. Phylogenetic analysis showed that RD-5 strain was closely related to novel *Actinobacteria* bacterium such as TDI19 (KT021825), *S. radiopugnans* strain HBUM174026 (EU841544), *Streptomyces* sp. RC 1832 (JQ862603), *S. nanhaiensis* strain SCSIO 01248 (NR_108633), isolated from different geographical location including deep-sea sediment (Tian et al., 2012).

PCR amplification and identification of these biosynthetic genes was very important for assessing its potential for both culturable and unculturable microorganism (Minowa et al., 2007). Large numbers of biologically active compounds are identifying which is encoded by a set of genes, in which PKS-I and PKS-II are responsible for the biosynthesis of the active metabolite (Ayuso-Sacido and Genilloud, 2005). The presence of types I and II PKS gene in *S. variabilis* RD-5 showed a direct correlation with the identified bioactive compound, which is polyketide in nature.

The extracted compound of *S. variabilis* RD-5 was found the most active against pathogenic bacteria, and thus it can play an important role in clinical appliances. Extracellular enzymes play a key role in the recycling of organic carbon and nitrogen compounds in biotechnology. The strain RD-5 exhibited highest antibacterial activity against *Klebsiella pneumonia*. The result showed that secondary metabolite active compounds containing antibacterial activities were extracellular and it could be extracted, quantified and further explored for the discovery of new drugs (Passari et al., 2015). To best of our knowledge, this is the first report of *S. variabilis* RD-5 having strong antimicrobial activity against bacteria. From the results, we concluded that the results of morphological, biochemical characteristics and polyphasic approach; the isolate *S. variabilis* RD-5 was the member of *Actinobacteria*, which secretes bioactivity with novel characteristics.

The crude extract was tested and found good antioxidant properties which can be useful for further research development to make it the industrially important. The radical scavenging activity of the extract was concentration dependent, and gradual increase of concentration increased the activity which was supported by the report of Kumaqai et al. (1993). The DPPH free radical scavenging assay was extensively used to measure antioxidant capacity. Antioxidants react with DPPH and reduce the DPPH molecules equal to the number of freely available hydroxyl groups (Matthäus, 2002). The DPPH scavenging activity depends on the degree of due to its ability to donate hydrogen proton. With the same concentration, the isolate was capable of reducing Fe^3+^ ions which indicated the presence of active compounds in the solvent extracts (Kekuda et al., 2010). *S. variabilis* RD-5 is potential sources of antioxidants, which reflects by high hydrogen peroxide activity, is useful in preventing the progress of various oxidative stress-related disorders (Poongodi et al., 2012). Hydrogen peroxide has ability to cross cell membrane easily and also reacts with metal ions (Fe^2+^ and/or Cu^2+^) to produce ROS (reactive oxygen species) such as hydroxyl free radical which have toxic effects (Swant et al., 2009). Thus, the present study suggests that the Actinobacterial extract of RD-5 can act as better antioxidant agents for removing H_2_O_2_.

According to a report of Thenmozhi and Kannabiran (2012), ethyl acetate extract of *Streptomyces* species VITSTK7, isolated from marine environment of the Bay of Bengal, exhibited 43.2% DPPH scavenging activity and 51% metal chelating activity at 10 mg/mL concentration. Similarly, Karthik et al. (2013) reported antioxidant activity of three marine *Actinobacteria* isolated from marine sediments of Nicobar Islands whereas phenolic compounds extracted from *Streptomyces* sp. LK-3 exhibited 76% DPPH scavenging activity at 100 μg/mL. Two phenolic compounds from *Streptomyces* sp. JBIR-94 and JBIR-125 showed DPPH scavenging activity with an IC value of 11.4 and 35.1 μM, respectively. Sowndhararajan and Kang (2013) studied free radical scavenging potential of culture filtrate of *Streptomyces* sp. AM-S1 isolated from forest humus soil in Gyeongsan, South Korea where ethyl acetate extract exhibited higher activity as compared to the lyophilised cell-free supernatant. According to Rao and Rao (2013), the extracts of *Actinobacteria* isolated from mangrove soil of Vishakhapatnam region showed 46–70% DPPH scavenging activity and 68–78% FRAP activity at 20 μg/mL concentration. Karthik et al. (2014) reported an extracellular protease produced by a marine *Streptomyces* sp. MAB 18 which exhibited antioxidant activity. *Nocardiopsis alba* isolated from mangrove soil collected from Andhra Pradesh, India, exhibited antioxidant activity. The potential fraction obtained by chromatography showed antioxidant activity at par with standard ascorbic acid (Janardhan et al., 2014). Nagaseshu et al. (2016) reported antioxidant activity of methanol extracts of *Actinobacteria* isolated from marine sediment collected from Kakinada coast. They also correlated the antioxidant activity of the extract which cytotoxic and antiproliferative activities.

The results of FAME of carbon chain length C15–C17 is consistent with the long carbon chain with saturated fatty acids which is used to produce phospholipids for *Streptomyces* cell membranes. Possibly *S. variabilis* RD-5 makes a triglyceride lyase which breaks bonds present between the carbon atoms, in resultant the FAMEs were generated (Lu et al., 2013). Further elucidation of genome sequences of strain RD-5 should be helpful for the investigation how the FAMEs were generated.

## Conclusion

Adaptation of marine microorganism has developed prodigious physiological and metabolic capacities to survive in a harsh condition that triggered them to synthesize different metabolites, which could not be produced by the terrestrial ones. In the present study, *S. variabilis* RD-5 was isolated from Gulf of Khambhat, Alang, Bhavnagar, and screened for its ability to produce the bioactive compound. The extracted compounds show good antibacterial and antioxidant properties. Extracellular enzymes play a key role in recycling of organic carbon and nitrogen compounds in biotechnology.

## Author Contributions

Conceived and designed the experiments: KM and AM. Performed the experiments: RD and RK. Analyzed the data: RD, RK, and AM. Secured the funds to support this research: KM and BJ. Wrote the paper: RD and RK.

## Conflict of Interest Statement

The authors declare that the research was conducted in the absence of any commercial or financial relationships that could be construed as a potential conflict of interest.
