# Supplementary material for: Antibacterial and Antioxidant Activities of Novel Actinobacteria Strain Isolated from Gulf of Khambhat, Gujarat
Source: Front Microbiol. 2017 Dec 7;8:2420. doi: 10.3389/fmicb.2017.02420 (PMC5725476; doi:10.3389/fmicb.2017.02420)
Supplement: Supplementary file 1 [file Table_1.DOCX]

Table S1. Substrate metabolism profiles of RD-5 with reference strains of *Actinobacteria* incubated in at 30°C for 96 h

| **No.** |  | **Sources** | **RD1** | **RD2** | **RD3** | **RD4** | **RD5** | **RD6** | **RD15** | **RD7** | **RD8** | **RD9** | **RD16** |
| --- | --- | --- | --- | --- | --- | --- | --- | --- | --- | --- | --- | --- | --- |
| **1** |  | Negative Control | - | - | - | - | - | - | - | - | - | - | - |
| **2** | Carbohydrate | Dextrin | + | + | + | + | + | + | + | + | + | + | + |
| **3** |  | D-Maltose | + | + | + | + | + | + | + | + | + | + | + |
| **4** |  | D-Trehalose | + | + | + | + | + | + | + | + | + | + | + |
| **5** |  | D-Cellobiose | + | + | + | + | + | + | + | + | + | + | + |
| **6** |  | Gentiobiose | + | + | + | + | + | + | + | + | + | + | + |
| **7** |  | Sucrose | + | + | + | + | + | + | + | + | + | + | + |
| **8** |  | D-Turanose | + | + | + | + | + | + | + | + | + | + | + |
| **9** |  | Stachyose | + | + | + | + | + | + | + | + | + | + | + |
| **10** |  | Positive Control | + | + | + | + | + | + | + | + | + | + | + |
| **11** | pH | pH 6 | + | + | + | + | + | + | + | + | + | + | + |
| **12** |  | pH 5 | - | - | - | - | - | - | - | - | + | + | - |
| **13** | Sugar | D-Raffinose | + | + | + | + | + | + | + | + | + | + | + |
| **14** |  | α-D-Lactose | + | + | + | + | + | + | + | + | + | + | + |
| **15** |  | D-Melibiose | + | + | + | + | + | + | + | + | + | + | + |
| **16** |  | β-Methyl-DGlucoside | + | + | + | + | + | + | + | + | + | + | + |
| **17** |  | D-Salicin | + | + | + | + | + | + | + | + | + | + | + |
| **18** |  | N-Acetyl-D-Glucosamine | + | + | + | + | + | + | + | + | + | + | + |
| **19** |  | N-Acetyl-β-D-Mannosamine | + | + | + | + | + | + | + | + | + | + | + |
| **20** |  | N-Acetyl-D-Galactosamine | + | + | + | + | + | + | + | + | + | + | + |
| **21** |  | N-Acetyl Neuraminic Acid | + | + | + | + | + | + | + | + | + | + | + |
| **22** | Salt concentration | 1% NaCl | + | + | + | + | + | + | + | + | + | + | + |
| **23** |  | 4% NaCl | + | + | + | + | + | + | + | + | + | + | + |
| **24** |  | 8% NaCl | + | - | - | + | + | - | - | + | + | + | + |
| **25** | Sugar | α-D-Glucose | + | + | + | + | + | + | + | + | + | + | + |
| **26** |  | D-Mannose | + | + | + | + | + | + | + | + | + | + | + |
| **27** |  | D-Fructose | + | + | + | + | + | + | + | + | + | + | + |
| **28** |  | D-Galactose | + | + | + | + | + | + | + | + | + | + | + |
| **29** |  | 3-Methyl Glucose | + | + | + | + | + | + | + | + | + | + | + |
| **30** |  | D-Fucose | + | + | + | + | + | + | + | + | + | + | + |
| **31** |  | L-Fucose | + | + | + | + | + | + | + | + | + | + | + |
| **32** |  | L-Rhamnose | + | + | + | + | + | + | + | + | + | + | + |
| **33** |  | Inosine | + | + | + | + | + | + | + | + | + | + | + |
| **34** | Salt concentration | 1% Sodium Lactate | + | + | + | + | + | + | + | + | + | + | + |
| **35** | Antibiotic | Fusidic Acid | - | - | - | - | - | + | - | - | - | + | - |
| **36** | Aminoacid | D-Serine | - | - | - | + | - | + | - | + | + | - | + |
| **37** | Sugar | D-Sorbitol | + | + | + | + | + | + | + | + | + | - | + |
| **38** |  | D-Mannitol | + | + | + | + | + | + | + | + | + | + | + |
| **39** |  | D-Arabitol | + | + | + | + | + | + | + | + | + | + | + |
| **40** |  | myo-Inositol | + | + | + | + | + | + | + | + | + | + | + |
| **41** |  | Glycerol | + | + | + | + | + | + | + | + | + | + | + |
| **42** |  | D-Glucose- 6-PO4 | + | + | + | + | + | + | + | + | + | + | + |
| **43** |  | D-Fructose- 6-PO4 | + | + | + | + | + | + | + | + | + | + | + |
| **44** | Aminoacid | D-Aspartic Acid | + | + | + | + | + | + | + | + | + | + | + |
| **45** |  | D-Serine | - | - | - | + | + | + | - | + | + | + | + |
| **46** | Antibiotic | Troleandomycin | - | + | - | + | - | + | - | - | - | + | + |
| **47** |  | Rifamycin SV | + | + | + | + | + | + | + | + | + | - | + |
| **48** |  | Minocycline | - | - | - | - | + | + | - | - | - | + | - |
| **49** | Protein | Gelatin | + | + | + | + | + | + | + | + | + | - | + |
| **50** | Aminoacid | Glycyl-L-Proline | + | + | + | + | + | + | + | + | + | + | + |
| **51** |  | L-Alanine | + | + | + | + | + | + | + | + | + | + | + |
| **52** |  | L-Arginine | + | + | + | + | + | + | + | + | + | + | + |
| **53** |  | L-Aspartic Acid | + | + | + | + | + | + | + | + | + | + | + |
| **54** |  | L-Glutamic Acid | + | + | + | + | + | + | + | + | + | + | + |
| **55** |  | L-Histidine | + | + | + | + | + | + | + | + | + | + | + |
| **56** |  | L-Pyroglutamic Acid | + | + | + | + | + | + | + | + | + | + | + |
| **57** |  | L-Serine | + | + | + | + | + | + | + | + | + | + | + |
| **58** | Antibiotic | Lincomycin | - | - | - | - | + | + | - | - | - | + | - |
| **59** | Guanidine HCl | Guanidine HCl | + | - | - | + | + | + | + | + | + | - | + |
| **60** | Surface-active agent | Niaproof 4 | - | - | - | - | + | - | - | - | - | + | - |
| **61** | Polysaccharide | Pectin | + | + | + | + | + | + | + | + | + | - | + |
| **62** | Sugar | D-Galacturonic Acid | + | + | + | + | + | + | + | + | + | + | + |
| **63** | L-Galactonic Acid Lactone | L-Galactonic Acid Lactone | + | + | + | + | + | + | + | + | + | + | + |
| **64** | D-Gluconic Acid | D-Gluconic Acid | + | + | + | + | + | + | + | + | + | + | + |
| **65** | Uronic acid | D-Glucuronic Acid | + | + | + | + | + | + | + | + | + | + | + |
| **66** |  | Glucuronamide | + | + | + | + | + | + | + | + | + | + | + |
| **67** | Mucic Acid | Mucic Acid | + | + | + | + | + | + | + | + | + | + | + |
| **68** | Quinic Acid | Quinic Acid | + | + | + | + | + | + | + | + | + | + | + |
| **69** | D-Saccharic Acid | D-Saccharic Acid | + | + | + | + | + | + | + | + | + | + | + |
| **70** | Antibiotic | Vancomycin | - | - | - | - | + | + | - | - | + | + | - |
| **71** | Redox indicator | Tetrazolium Violet | + | - | + | + | + | - | + | + | + | - | + |
| **72** | Indicator | Tetrazolium Blue | + | - | + | + | + | + | + | + | + | + | + |
| **73** | Chemical indicator | p-HydroxyPhenylacetic Acid | + | + | + | + | + | - | + | + | + | + | + |
| **74** | Amino acid | Methyl Pyruvate | + | + | + | + | + | + | + | + | + | + | + |
| **75** |  | D-Lactic Acid Methyl Ester | + | + | + | + | + | + | + | + | + | + | + |
| **76** |  | L-Lactic Acid | + | + | + | + | + | + | + | + | + | + | + |
| **77** |  | Citric Acid | + | + | + | + | + | + | - | + | + | + | + |
| **78** |  | α-Keto-Glutaric Acid | + | + | + | + | + | - | + | + | + | + | + |
| **79** |  | D-Malic Acid | + | + | + | + | + | + | + | + | + | + | + |
| **80** |  | L-Malic Acid | + | + | + | + | + | + | + | + | + | + | + |
| **81** |  | Bromo-Succinic Acid | + | + | + | + | + | + | - | + | + | + | + |
| **82** | Antibiotic | Nalidixic Acid | + | + | + | + | + | + | + | + | + | + | + |
| **83** | Amino acid | Lithium Chloride | + | + | + | + | + | + | + | + | + | + | + |
| **84** |  | Potassium Tellurite | + | + | + | + | + | + | + | + | + | + | + |
| **85** |  | Tween 40 | + | + | + | + | + | + | + | + | + | + | + |
| **86** |  | γ-Amino-Butryric Acid | + | + | + | + | + | + | + | + | + | + | + |
| **87** |  | α-HydroxyButyric Acid | + | + | + | + | + | + | + | - | + | + | + |
| **88** |  | β-Hydroxy-D,LButyric Acid | + | + | + | + | + | + | + | + | + | + | + |
| **89** |  | α-Keto-Butyric Acid | + | + | + | + | + | + | + | + | + | + | + |
| **90** |  | Acetoacetic Acid | + | + | + | + | + | + | + | + | + | + | + |
| **91** |  | Propionic Acid | + | + | + | + | + | + | + | + | + | + | + |
| **92** |  | Acetic Acid | + | + | + | + | + | + | + | + | + | + | + |
| **93** |  | Formic Acid | + | + | + | + | + | + | + | + | + | + | + |
| **94** | Antibiotic | Aztreonam | + | + | + | + | + | + | + | + | + | + | + |
| **95** | Salt concentration | Sodium Butyrate | + | + | - | + | + | + | + | + | + | + | + |
| **96** |  | Sodium Bromate | + | + | - | + | + | + | - | + | + | + | + |
